# Efficacy of using Maryland forceps versus electrocoagulation hooks in da Vinci robot-assisted thoracoscopic mediastinal tumor resection

**DOI:** 10.1186/s12957-023-03065-y

**Published:** 2023-06-19

**Authors:** Ziqiang Hong, Xiangdou Bai, Yannan Sheng, Baiqiang Cui, Yingjie Lu, Tao Cheng, Xusheng Wu, Dacheng Jin, Yunjiu Gou, Jing Zhao

**Affiliations:** 1 The First Clinical Medical College of Gansu University of Chinese Medicine, Gansu Provincial Hospital, Lanzhou, China; 2 Department of Thoracic Surgery, Gansu Provincial Hospital, Lanzhou, China; 3 Lanzhou First People’s Hospital, Lanzhou, China

**Keywords:** Robot-assisted thoracic surgery, Mediastinal tumors, Maryland forceps, Electrocoagulation hooks

## Abstract

**Background:**

To compare the difference of short-term curative effect between the use of Maryland forceps (MF) and electrocoagulation hooks (EH) in da Vinci robot-assisted thoracoscopic mediastinal tumor resection.

**Methods:**

Retrospectively analyze 84 patients with mediastinal tumors who underwent robot-assisted thoracoscopic surgery (RATS) at the Department of Thoracic Surgery in Gansu Provincial Hospital from February 2019 to February 2023. Two groups were divided according to the intraoperative use of energy devices, including 41 cases in the MF group and 43 cases in the EH group. Perioperative clinical data was gathered to compare the short-term efficacy of patients in both groups.

**Results:**

There were no significant differences in baseline characteristics such as sex (*P* = 0.685), age (*P* = 0.165), and tumor size (*P* = 0.339) between the two groups. Compared with the EH group, patients in the MF group have shorter operative time (*P* = 0.030), less intraoperative bleeding (*P* = 0.010), less total postoperative drainage volume (*P* = 0.001), shorter postoperative drainage time (*P* = 0.022), shorter hospital stay (*P* = 0.019), and lower levels of interleukin-6 (IL-6), interleukin-8 (IL-8), tumor necrosis factor-alpha (TNF-α), and cortisol. No statistically significant differences were found between the two groups in terms of total hospitalization costs (*P* = 0.123), postoperative visual analog scale (VAS) pain scores (*P* = 0.064), and postoperative complications (*P* = 0.431).

**Conclusion:**

Using MF in RATS for mediastinal tumor is safe and effective, which can reduce the amount of bleeding, reduce the degree of inflammatory reaction, and conducive to the quick recovery of patients.

## Introduction

Mediastinal tumors are typically benign, and patients often remain asymptomatic in the early stages. They are usually detected through physical examination. As the tumors grow, they may exert pressure on surrounding tissues and organs, leading to compression symptoms. In such cases, surgical intervention commonly employed as a clinical treatment option [1, 2]. With the continuous development of minimally invasive technology, the minimally invasive surgical operation system represented by robot-assisted thoracic surgery (RATS) has been widely used in mediastinal tumor resection [3–5]. Two most commonly used energy instruments in current robotic surgical systems are electrocoagulation hooks (EH) and Maryland forceps (MF). Single-stage EH is widely employed due to its simple structure and mature technology. However, in recent years, some centers have turned to the application of dual-stage MF due to its ability to reduce thermal damage to adjacent tissues while still meeting the requirements for more refined anatomical separation than EH [6, 7]. However, some scholars believed that the use of MF is controversial because of the fear of poor perioperative efficacy due to excessive tearing and pulling movements during the use of MF. Therefore, in this paper, we retrospectively analyzed the clinical data of patients in the intraoperative EH and MF groups to compare the difference in short-term efficacy between the use of MF and EH in robotic-assisted thoracoscopic mediastinal tumor resection.

## Materials and methods

### Clinical information

This study is a retrospective cohort study. The research analyzed clinical data from 84 patients suffered with mediastinal tumors who received RATS treatment at the Department of Thoracic Surgery in Gansu Provincial Hospital from February 2019 to February 2023. Inclusion criteria are as follows: (1) the mediastinal tumor was confirmed by contrast enhanced computed tomography (CT) before the operation [8]; (2) the imaging suggests that the lesion is non-invasive, with clear boundaries, no obvious invasion of surrounding tissues or organs, no involvement of major blood vessels, and no distant metastasis; (3) preoperative cardiopulmonary function should be generally normal, with no serious complications, no history of other thoracic surgeries, and no relevant history of conditions such as tuberculosis or empyema that could cause extensive adhesions in the chest cavity.

Exclusion criteria are as follows: (1) simultaneous surgery for mediastinal tumors combined with lung disease and (2) patients who underwent radiotherapy and chemotherapy before surgery.

All patients included in the study were operated by the same surgeon during the same stage. The study was reviewed by the Ethics Committee of Gansu Provincial Hospital, approval number: 2023–231.

### Preoperative preparation

Preoperatively, patients in both groups underwent respiratory function training of equal intensity. Routine preoperative examinations, including electrocardiogram, cardiac and digestive system ultrasound, pulmonary function and chest CT scans, complete blood count, basic metabolic panel, coagulation studies, eight-item preoperative infection screening, blood typing, and tumor marker evaluation were performed to identify any contraindications for surgery. Patients with preoperative myasthenia gravis symptoms were diagnosed with myasthenia gravis by neurology consultation, electromyography, and neostigmine test and were treated with oral pyridostigmine 2 weeks before surgery.

### Surgery methods

**Fig. 1:**
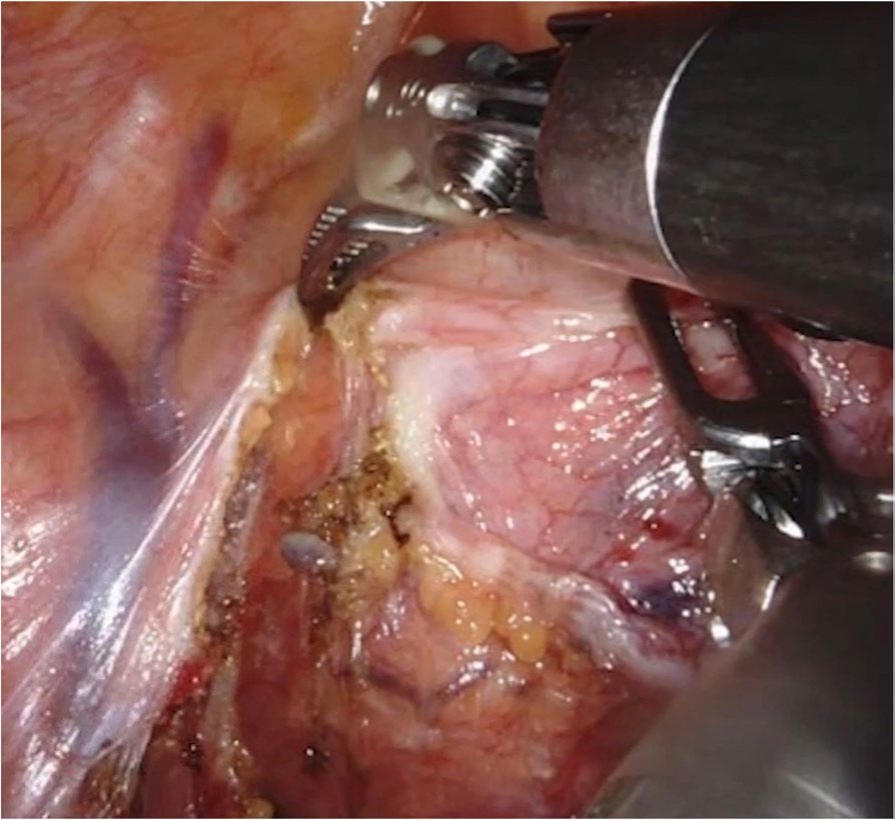
Dissection of mediastinal tumors with Maryland forceps

**Fig. 2:**
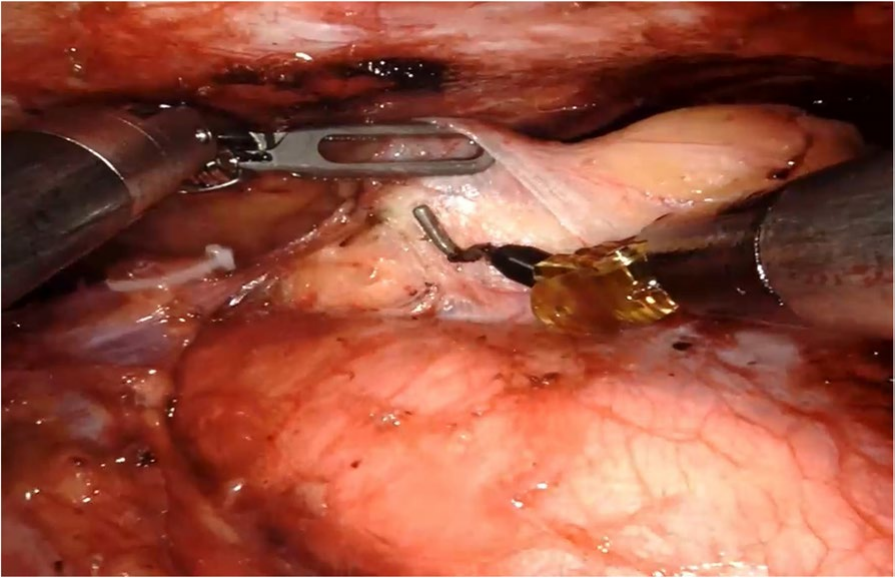
Dissection of mediastinal tumors with electrocoagulation hooks

During the procedure, the patient was given general anesthesia and a laryngeal mask ventilation was used. An artificial pneumothorax was created by accessing CO_2_ at a pressure of 6–8 mm Hg (1 mm Hg = 0.133 kPa) [8]. Position: generally, the upper body of the operated side can be elevated 30 to 45° in a semi-supine position (the upper limb of the affected side is abducted to expose the axilla and fixed on the anesthesia frame), etc. [8]. The hole is set in the “5–3-5” method, with “5” being the observation hole in the fifth intercostal space of the anterior axillary line on the affected side, “3” being the operation hole of arm 1 in the third intercostal space of the anterior axillary line, and “5” being the operation hole of arm 2 in the fifth intercostal space of the midclavicular line [8]. The left hand was a non-injury grasping clamp, and only the right hand energy instruments were different between the EH and MF groups, while the rest was identical (Figs. 1 and 2). After exploring the thoracic cavity, the mediastinal pleura around the tumor is cut with a combination of energy instruments, using the phrenic nerve, internal mammary vein, unnamed vein, sternum, and pericardium as the boundary. Afterwards, carefully distinguishing the dissection, freeing the mass, identifying and protecting the phrenic nerve, for the larger trophoblastic vessels need to be completely free. The blood vessels are occluded using Hem-o-lock or titanium clips, followed by cutting them with MF or EH [8]. It was important to ensure adequate visualization of the left ring vein during surgery to avoid injury. For thymomas, etc., a total thymectomy is required (if combined with severe myasthenia gravis, anterior mediastinal fat removal is added) [8]. When the specimen is removed, the operation hole size can be extended appropriately.

### Observed indicators

Patient’s sex, age, body mass index (BMI), smoking history, tumor size, tumor location, and tumor type are all important preoperative information. Intraoperative information included operative time and intraoperative bleeding, and postoperative data included total postoperative drainage volume, postoperative drainage time, postoperative hospital stay, total cost of hospitalization, visual analog scale (VAS) pain score, postoperative complications, and postoperative changes in c-reactive protein (CRP), interleukin-6 (IL-6), interleukin-8 (IL-8), cortisol, and tumor necrosis factor-alpha (TNF-α).

### Statistical analyses

We used SPSS 26.0 software for statistical analysis. Continuous variables were expressed as mean ± standard deviation ($\stackrel{\mathrm{-}}{\text{x}}$±s). A *t* test was used to compare two independent samples, while the chi-square or Fisher’s exact test was used to compare categorical variables between groups. Statistical significance was defined as a *P* value less than 0.05.

## Results

### Perioperative conditions

There were no statistically significant differences between the two groups in baseline indicators such as sex (*P* = 0.685), age (*P* = 0.165), and tumor size (*P* = 0.339), as summarized in Table 1.

**Table 1:** Comparison of baseline information between the two groups [cases (%)/$\stackrel{\mathrm{-}}{\text{x}}$±*s*]

Characteristic	MF group (*n* = 41)	EH group (*n* = 43)	*P* value
Sex			0.685
Male	23 (56.1)	26 (60.5)	
Female	18 (43.9)	17 (39.5)	
Age (years)	45.66 ± 4.68	47.21 ± 5.41	0.165
BMI (kg/m^2^)	23.46 ± 2.40	24.02 ± 2.30	0.278
Smoking history			0.696
Yes	9 (22.0)	11 (25.6)	
No	32 (78.0)	32 (74.4)	
Tumor size (mm)	38.73 ± 5.65	39.88 ± 5.33	0.339
Tumor location			0.884
Anterior	32 (78.0)	34 (79.1)	
Middle	6 (14.6)	5 (11.6)	
Posterior	3 (7.4)	4 (9.3)	
Tumor type			0.853
Benign cyst	19 (46.3)	22 (51.2)	
Thymoma	13 (31.7)	10 (23.3)	
Thymic hyperplasia	6 (14.6)	7 (16.2)	
Neurogenic tumor	3 (7.4)	4 (9.3)	

Compared with the EH group, patients in the MF group have shorter operative time (*P* = 0.030), less intraoperative bleeding volume (*P* = 0.010), less total postoperative drainage volume (*P* = 0.001), shorter postoperative drainage time (*P* = 0.022), and shorter postoperative hospital stay (*P* = 0.019). And no significant differences were detected between the two groups in total cost of hospitalization (*P* = 0.123), postoperative VAS pain scores (*P* = 0.064), and postoperative complications (*P* = 0.431); see Table 2 for details.

**Table 2:** Comparison of intraoperative and postoperative indexes between the two groups [cases (%)/$\stackrel{\mathrm{-}}{\text{x}}$±*s*]

Characteristic	MF group (*n* = 41)	EH group (*n* = 43)	*P* value
Operating time (min)	98.66 ± 17.25	110.35 ± 17.57	0.030
Intraoperative bleeding volume (ml)	28.66 ± 9.29	35.58 ± 8.68	0.010
Total postoperative drainage volume (ml)	184.27 ± 25.46	203.14 ± 18.19	0.001
Postoperative drainage time (days)	3.05 ± 0.84	3.60 ± 1.12	0.022
Postoperative hospital stay (days)	4.63 ± 0.89	5.16 ± 0.84	0.019
Total cost of hospitalization ($)	7562.53 ± 538.91	7388.35 ± 483.79	0.123
Postoperative VAS pain score			
12 h after surgery	3.31 ± 0.93	3.59 ± 0.88	0.161
24 h after surgery	2.90 ± 0.80	3.16 ± 0.72	0.121
48 h after surgery	1.54 ± 0.75	1.84 ± 0.72	0.064
Postoperative complications	2 (4.8)	4 (9.3)	0.431
Arrhythmia	1 (2.4)	2 (4.7)	
Pleural effusion	0	1 (2.3)	
Pulmonary infection	1 (2.4)	0	
Pulmonary atelectasis	0	1 (2.3)	

*Abbreviation*: *VAS* visual analog scale, *$* dollars

There were no statistically significant differences in the levels of CRP, IL-6, IL-8, Cortisol, and TNF-α between the MF and EH groups 1 day before surgery. The levels of IL-6, IL-8, cortisol, and TNF-α in the MF group were lower than those in the EH group on the first day after surgery, as showed in Table 3.

**Table 3:** Comparison of blood CRP, IL-6, IL-8, cortisol, and TNF-α indexes between two groups of patients ($\stackrel{\mathrm{-}}{\text{x}}$±*s*)

Factors	Time	MF group (*n* = 41)	EH group (*n* = 43)	*P* value
CRP (mg/L)	One day before surgery	3.10 ± 0.46	2.99 ± 0.39	0.230
	One day after surgery	9.22 ± 1.28	9.92 ± 1.18	0.101
IL-6 (pg/ml)	One day before surgery	94.96 ± 9.78	98.17 ± 14.79	0.246
	One day after surgery	188.68 ± 19.31	210.75 ± 19.05	0.013
IL-8 (pg/ml)	One day before surgery	0.50 ± 0.08	0.52 ± 0.07	0.234
	One day after surgery	2.06 ± 0.69	2.39 ± 0.59	0.024
Cortisol (ng/ml)	One day before surgery	224.92 ± 17.05	227.32 ± 22.08	0.579
	One day after surgery	339.62 ± 24.92	366.11 ± 25.96	0.016
TNF-α (pg/ml)	One day before surgery	2.17 ± 0.62	2.07 ± 0.64	0.467
	One day after surgery	14.03 ± 2.64	15.71 ± 3.04	0.019

## Discussion

As a common disease in thoracic surgery, the surgical treatment of mediastinal lesions underwent significant changes in recent years, mainly due to the popularity of minimally invasive surgery and the rapid development of energy platforms. Compared with the thoracoscopic surgery, robotic surgical systems have better visualization and more flexible instrumentation, so they have also been widely used in the treatment of mediastinal tumors in recent years [9, 10]. Two main types of energy instruments in current robotic surgical systems are MF and EH. There are two primary types of energy instruments used in current robotic surgical systems: MF and EH. Robotic surgeons performing thoracic surgeries have transitioned to learning based on skilled thoracoscopic operations and are already familiar with the use of EH during thoracoscopy. Consequently, it is logical that EH became the preferred energy separation device for robotic surgery in each center. However, in recent years, the concept of refined and tubeless surgery gradually emerged due to the requirement of rapid postoperative recovery. Therefore, coarse, blunt, and thermally damaged EH cannot meet the requirements of robotic surgery. Consequently, medical centers are gradually replacing these instruments with bipolar MF [11, 12]. Among them, urology and gynecology have pioneered the use of MF with bipolar scissors in robotic surgery, which showed unique advantages in vascular freeing and lymph node dissection [13]. Since the introduction of thoracic robotic surgery into MF, there have been mixed reviews from scholars, and no relevant data have been reported in the literature. Therefore, a detailed comparison of the perioperative efficacy of the two energy devices were performed in our center, with the hope of providing some insight into the selection of energy devices.

EH and MF are commonly used energy instruments in thoracic robotic surgery, with different indications and precautions based on their working principles. EH employs monopolar electrocoagulation to generate high temperatures, leading to carbonization and crusting of the target tissue. Its primary action is pulling back, making it suitable for use when there are critical structures, such as blood vessels, in front of the operative field. MF refers to bipolar electrocoagulation, which features a tip that allows for precise spot coagulation and can be utilized for clamping, stitching, ligating, separating, and coagulating during intraoperative procedures. Our study found that the MF group has shorter operative times, less intraoperative bleeding, and shorter postoperative hospital stays compared with the EH group. Compared with thoracoscopic EH, robotic EH is more coarse and blunt and less invasive. Even with the multi-joint advantage of the robotic system, there are limitations to the use of robotic EH in mediastinal tumor resection. On the basis of the traditional “hook, block, dial, and winding” operation, it is difficult to play the advantages of the robot arm. The grasping pliers with MF “double pliers” mode of operation is more ergonomic, but also better free the right hand to open and close the multi-angle operation. On the basis of “hook, block, poke, and wrap” to achieve the operation of clamping, ligation, and blunt separation, etc. The MF has a slimmer tip, making it easier to dissect the finer vessels compared with the EH. MF is not only used for dissection, but also can be used with non-invasive grasping forceps to complete ligation and knotting or even simple suturing. MF can also cut blood vessels, trachea, and even sutures, and there is almost no need to change other instruments during the operation, which can reduce the cost and time of surgery accordingly. In addition, local high-temperature electrocoagulation of EH can easily generate smoke, affecting the surgical field and possibly influencing the surgical process.

Due to the special location of the thymus gland and the invasion of some thymomas, the pericardium is invaded in some patients, and a portion of the pericardium can be lifted directly using Maryland to give excision, separation, and hemostasis. After removing part of the pericardium and clearing the prepericardial fat, the phrenic nerve continues to free upward along the anterior border of the phrenic nerve, on the right side to the point where the internal thoracic vein converges into the superior vena cava and on the left side to the point where the internal thoracic vein converges into the left innominate vein, where the tip of the MF point coagulation can well protect the phrenic nerve and prevent the patient from postoperative respiratory paralysis and diaphragm elevation. Our study disclosed that although there was no significant difference in the postoperative complication rate between the two groups, the MF group has lower complication rate. This is due to the smaller energy spread of MF bipolar electrocoagulation, and the refinement of freeing based on precise spot coagulation also greatly reduces intraoperative nerve damage. Additionally, the reduced energy output resulted in less postoperative drainage, shorter postoperative hospital stay, and decreased patient burden, aligning with the principles of “precise and minimally invasive” surgery.

Surgical stimulation can lead to the proliferation of peripheral blood vessels, promote the release of inflammatory factors, affect surrounding tissues and organs, and be detrimental to postoperative recovery [14]. TNF-α is mainly secreted by activated monocyte B macrophages and is involved in immune regulation and inflammatory responses in the body [15]. CRP and IL-6 are all pro-inflammatory factors, and persistent elevation of postoperative inflammatory factor levels may cause thrombosis or even affect organ function. IL-8 activates neutrophils, which in turn mediate the inflammatory response of the body. The level of cortisol is an index to evaluate the degree of immune stress response. The results of this study revealed that postoperative CRP, IL-6, IL-8, cortisol, and TNF-α indexes were higher in the MF and EH groups than those before surgery, and the increase in IL-6, IL-8, cortisol, and TNF-α in the MF group were less than those in the EH group. This indicates that MF patients have lower levels of inflammatory markers and a milder inflammatory state after treatment. The main reason is that MF is more delicate and has better minimally invasive characteristics, which is less traumatic to the patient as a whole, and the stress level of the patient’s organism is relatively lower, and the expression of inflammatory indexes is significantly reduced.

There are some limitations and shortcomings to this study: (1) small sample sizes in the study and a single-center data source may produce biased results; (2) including patients who underwent surgery by the same surgeon at the same stage in this study ignores the skill and experience differences between different surgeons. This may lead to bias in the conclusions; (3) this study compared only recent efficacy and survival outcomes and did not perform long-term survival analyses.

## Conclusion

In summary, the utilization of MF in robot-assisted thoracoscopic mediastinal tumor resection is both safe and effective and has significant advantages in reducing operative time, intraoperative bleeding, and surgical trauma. However, due to the limited sample size of the studies included, further clinical trials and long-term follow-up are essential to confirm its long-term efficacy and provide more precise guidance for clinical application.

## Data Availability

The datasets used and/or analyzed during the current study are available from the corresponding author on reasonable request.

## Abbreviations

MF: Maryland forceps
EH: Electrocoagulation hooks
RATS: Robot-assisted thoracic surgery
CT: Computed tomography
BMI: Body mass index
VAS: Visual analog scale
$: Dollars
CRP: C-reactive protein
IL-6: Interleukin-6
IL-8: Interleukin-8
TNF-α: Tumor necrosis factor-alpha
